# Comparison of Oral Health Status and Knowledge on Oral Health in Two Age Groups of Schoolchildren: A Cross-sectional Study

**DOI:** 10.5005/jp-journals-10005-1462

**Published:** 2017-02-27

**Authors:** PR Geethapriya, Sharath Asokan, D Kandaswamy

**Affiliations:** 1 Professor, Department of Pediatric Dentistry, K.S.R. Institute of Dental Science and Research, Tiruchengode, Tamil Nadu, India; 2 Professor and Head, Department of Pediatric Dentistry, K.S.R. Institute of Dental Science and Research, Tiruchengode, Tamil Nadu, India; 3 Dean, Professor and Head, Department of Conservative Dentistry, Sri Ramachandra Dental College and Hospital, Chennai, Tamil Nadu, India

**Keywords:** Caries treatment needs, Cross sectional study Oral health, Schoolchildren.

## Abstract

**Introduction:**

Oral health plays a pivotal role in the overall wellbeing of children. As children grow, there is a variation in their oral health status due to the changing trends and lifestyle.

**Aim:**

To evaluate and compare the factors related to oral health status in two age groups of school going children.

**Materials and methods:**

A total of 582 children aged 8 to 11 years from 3 schools were included. Based on their school grade, they were grouped as younger (third grade) and older (fifth grade) children. Their dental caries status, caries treatment needs, oral hygiene status were assessed. A questionnaire was given to them to assess their knowledge on oral health.

**Results:**

Both third and fifth grade children had similar caries status. The caries treatment needs was significantly higher (p = 0.02) in fifth grade children of school III. The oral hygiene status was significantly better (p = 0.004) in fifth grade children of school I and third grade children (p < 0.001) of school III. Fifth grade children were found to have more knowledge on oral health and it was statistically significant in school II (p = 0.001). In school III, as caries status increased, the oral hygiene index score significantly increased (p = 0.001).

**Conclusion:**

Age did not have any influence on the oral health status of children. The older children had better knowledge on oral health, but the oral hygiene practices were not followed effectively

**How to cite this article:** Geethapriya PR, Asokan S, Kandaswamy D. Comparison of Oral Health Status and Knowledge on Oral Health in Two Age Groups of Schoolchildren: A Cross-sectional Study. Int J Clin Pediatr Dent 2017;10(4): 340-345.

## INTRODUCTION

Childhood is a distinct phase of life. Nature and nurture play a major role in the changes that happen in the physical, cognitive, emotional, and psychosocial development of children. The oral health status of a child greatly influences all these domains of growth and development. Oral health is an essential part of general health. Like any other disease, oral diseases also have psychosocial and emotional consequences apart from the biological effects.^1^ Children with poor oral health have 12 times more risk of having missed school days and restricted activity.^2^

As children grow, the prevalence of dental caries increases^3^ and the pattern of occurrence of caries also changes. In primary dentition, the occlusal surface is the most susceptible surface for dental caries. But, in mixed dentition, as contact areas get established, an increase in the incidence of proximal caries is noted.^4^ Maintenance of oral hygiene becomes crucial and challenging as children grow. According to the American Academy of Pediatric Dentistry, toothbrushing should be supervised by the parents/caregivers at least until the age of 8 years. Then, there is a shift from parental guidance in oral health maintenance to personal care. Research on the oral health status of children based on age has shown varied results across the globe and within different parts of India. Kundu et al^5^ analyzed various studies carried out across the country and found the prevalence of dental caries to be the highest in 15-year-olds, followed by 5- and 12-year-olds (62.02, 48.11, and 43.34% respectively). Lack of knowledge has been considered as an obstacle for improvement of oral health.

Knowledge related to oral health has been accepted as a prerequisite for health-related behavior. Research has shown that children with good oral health habits had high knowledge on oral health.^6^ As the age of the child increases, the level of its knowledge on oral health is expected to increase from home, school, and social experiences. Age seems to have an influence on the oral health status and the knowledge of children on oral health. Hence, the present study was planned to evaluate and compare the factors related to oral health status in two age groups of school-going children in Tiruchengode, South India.

## MATERIALS AND METHODS

The protocol of this cross-sectional study was approved by the Institutional Review Board and Institutional Ethics Committee of K.S.R. Institute of Dental Science and Research, Tiruchengode, Tamil Nadu, India. Three private schools (I, II, III) in Tiruchengode were selected based on the following criteria: (a) Same socioeconomic status (SES); (b) same syllabus; (c) same language medium; and (d) same locality of residence. Legal permission from concerned authorities of all the three schools was obtained, after explaining the purpose of the study. Children aged 8 to 11 years, belonging to Jean Piaget’s concrete operational period, were screened. They were divided into two groups: younger (third grade) and older children (fifth grade). All healthy children with informed written consent from their parents were included in this study. They were screened to assess their dental caries status (dft and mean number of decayed, missing, or filled teeth index), caries treatment needs using World Health Organization criteria (1997), and oral hygiene status using Oral Hygiene Index-Simplified (OHI-S). All children were screened by the primary investigator to avoid interobserver variability and bias.

A close-ended questionnaire was designed to assess the knowledge of children on oral health. This questionnaire was adapted from the research work of Al-Omiri et al.^7^ The questionnaire was also translated in the native language Tamil. A pilot trial was done to check the content and criteria validity of the questionnaire. Corrections were made and approved in a focus group discussion with eminent pediatric dentists. The final questionnaire had nine questions related to oral health. The cumulative score was recorded as the overall knowledge score. The questionnaire was filled by all the children in their respective classrooms in about 10 minutes. Questions focused on the frequency of brushing and snacking, effect of sugars and carbonated drinks on teeth, importance of tongue cleaning, bleeding gums, the time and importance of regular dental visit, and oral health and its relation to general health.

The data obtained were analyzed separately for each school using Statistical Package for the Social Sciences version 17 (Chicago, Illinois, USA). This will be used as the baseline data for the research planned to study the effect of different school dental health education interventions on oral health of children. Frequency distributions of the sample and data in the form of median (range) were tabulated. Based on the number of decayed teeth the children had, they were subgrouped into subgroup “a” (no caries), subgroup “b” (caries < 3), and subgroup “c” (caries > 3). Comparison of dental caries status, its treatment needs, and OHI-S and knowledge questionnaire scores between third- and fifth-grade children were done for the three schools separately using chi square appropriately. A p-value < 0.05 was considered statistically significant.

**Table 1:** **Table 1:** Comparison of oral health status variables between III- and V-grade children in three schools

		*School I median (range)*						*School II median (range)*						*School III median (range)*					
*Variables*		*III grade*		*V grade*		*p^a^-value*		*III grade*		*V grade*		*p^a^-value*		*III grade*		*V grade*		*p^a^-value*	
N		109		62				133		95				118		65					
Dental caries		0 (1.5)		0 (1.25)		0.98		1.00 (3)		0 (3)		0.41		0 (2.25)		0 (4)		0.28	
Caries treatment needs		1.00 (3)		0.50 (3)		0.63		2.00 (4)		1.00 (4)		0.19		0 (4)		2.00 (4)		0.02	
OHI-S		1.00 (0.83)		0.58 (0.83)		0.004		1.00 (0.5)		1.00 (0.83)		0.75		0.50 (0.83)		1.00 (0.84)		<0.001	
Overall knowledge		5 (6)		6 (6)		0.13		5 (6)		6 (5)		0.001		5 (7)		6 (6)		0.14	

^a^Chi-square test

## RESULTS

A total of 582 children participated in this study. Table 1 shows the sample distribution and comparison of oral health variables between third- and fifth-grade children of the three schools. The sample distribution of third-grade children (n = 360) in the three schools was as follows: school I (n = 109), school II (n = 133), and school III (n = 118). The fifth-grade children (n = 222) were distributed in school I (n = 62), school II (n = 95), and school III (n = 65). Comparison of the dental caries status between the two age groups showed no significant difference in all the three schools. In school III, there was a significant difference in the caries treatment needs (p = 0.02). There was a significant difference in the OHI-S scores in school I (p = 0.004) and school III (p < 0.001). School II showed a significant difference in the overall knowledge score (p = 0.001).

Table 2 shows the comparison of the OHI-S scores, caries treatment needs, and overall knowledge scores within the caries subgroups of third- and fifth-grade children in all three schools. The third-grade children of school III showed a significant difference (p = 0.001) in the OHI-S score between their caries subgroups. There was no significant difference in the OHI-S scores within the caries subgroups of fifth-grade children in all three schools. There was a statistically significant difference in the caries treatment needs between the caries subgroups of third- and fifth-grade children in all three schools. There was no significant difference in the overall knowledge scores between the caries subgroups in the three schools.

**Table 2:** **Table 2:** Comparison of OHI-S scores, caries treatment needs, and overall knowledge scores within caries subgroups of III- and V-grade children in all three schools

		*III-grade median (range)*								*V-grade median (range)*							
*Schools*		*Subgroup a*		*Subgroup b*		*Subgroup c*		*p^a^-value*		*Subgroup a*		*Subgroup b*		*Subgroup c*		*p^a^-value*	
*OHI-S scores*																	
School I		0.83 (0.83)		1.00 (0.92)		0.83 (0.66)		0.54		0.50 (0.58)		0.99 (1.26)		0.50 (0.84)		0.28	
School II		1.00 (0.5)		1.00 (0.71)		1.00 (0.37)		0.26		1.00 (1)		1.00 (0.66)		1.00 (0.33)		0.24	
School III		0.33 (0.83)		0.33 (0.5)		0.83 (0.66)		0.001		1.16 (1)		1.00 (0.83)		1.00 (0.96)		0.29	
*Caries treatment needs score*																	
School I		0 (0)		1.00 (2)		6.00 (3)		<0.001		0 (0)		1.50 (2)		5.00 (4)		<0.001	
School II		0 (0)		3.00 (2)		7.00 (4)		<0.001		0 (0)		2.00 (2)		4.00 (2)		<0.001	
School III		0 (0)		3.00 (1)		5.00 (3)		<0.001		0 (1)		2.00 (3)		6.00 (5)		<0.001	
*Overall knowledge score*																	
School I		5.00 (2)		5.50 (3)		5.00 (2.75)		0.710		6.00 (2.5)		5.50 (2.25)		6.00 (1)		0.540	
School II		5.00 (1)		6.00 (2)		5.00 (2.25)		0.81		6.00 (2)		6.00 (2)		6.00 (2)		0.52	
School III		5.00 (2)		6.00 (2)		5.00 (0.75)		0.16		6.00 (3)		6.00 (2)		5.00 (3)		0.320	

^a^Chi-square test

**Table 3:** **Table 3:** Comparison of OHI-S scores, caries treatment needs, and overall knowledge scores between III- and V-grade schoolchildren based on caries status

*III grade vs V grade*							
		*Subgroup a*		*Subgroup b*		*Subgroup c*	
*OHI-S^3^*							
School I		0.011		0.595		0.197	
School II		0.361		0.883		0.647	
School III		<0.001		0.002		0.188	
*Caries treatment needs^a^*							
School I		0.41		0.49		0.61	
School II		0.41		0.048		0.01	
School III		0.04		0.91		0.67	
*Overall knowledge^a^*							
School I		0.15		0.65		0.12	
School II		0.01		0.06		0.15	
School III		0.02		0.39		0.63	

^a^p-values calculated using chi-square test have been tabulated

Table 3 shows the comparison of OHI-S scores, caries treatment needs, and overall knowledge scores between third- and fifth-grade children, based on their caries status. In subgroup a of schools I and III, there was a significant difference (p = 0.011 and p < 0.001 respectively) in the OHI-S score. Subgroup “b” of school III showed a significant difference in the OHI-S score (p = 0.002). In school II, there was a significant difference in the caries treatment needs in subgroups “b” and “c” (p = 0.048, p = 0.01 respectively). Subgroup “a” of school III showed a significant difference (p = 0.04) in the caries treatment needs. Subgroup “a” of schools II and III showed a significant difference in the overall knowledge score (p = 0.01, p = 0.02 respectively).

Table 4 shows the distribution and comparison of responses for the knowledge questionnaire between third- and fifth-grade children in all the three schools. Responses between third- and fifth-grade children showed a significant difference for the questions related to effect of carbonated drinks and frequent snacking on teeth in school I (p < 0.001) and in school III (p = 0.014). In school II, there was a significantly different response for the question on the time for dental visit (p = 0.029). The question on bleeding gums received a significantly different response in all three schools (school I p = 0.043, school II p = 0.005, and school III p = 0.045). There was no significant difference in the responses given by third-and fifth-grade children to questions related to frequency of brushing, effect of sugars on dental tissues, tongue cleaning, and importance of mouth and oral health and its relation to general health (p > 0.05).

## DISCUSSION

This cross-sectional study comprehensively investigated the dental caries status, its treatment needs, oral hygiene status, and the knowledge on oral health of school children in Tiruchengode, South India. It also compared the factors related to oral health status in two age groups of school-going children. Jean Piaget’s concrete operational period includes children aged 7 to 11 years. The thinking process of these children is more scientific and systematic than younger children.^8^ They also have the mental cognition to understand and reply to the questions on oral health. Generally, as age increases, cognitive development also improves. But, it can differ in each child. Hence, two age groups of children belonging to the concrete operational period were included in the present study: Third-grade (8-9 years) and fifth-grade (10-11 years) children. All the children in this study belonged to private schools following the same syllabus, same language medium, and almost the same annual fees structure. Piovesan et al^9^ demonstrated that school type could be used as an alternative measure of SES and, hence, all the children in this study can be considered to belong to the same SES.

**Table 4:** **Table 4:** Distribution and comparison of responses for the knowledge questionnaire of III- and V-grade children in all three schools

		*School I*						*School II*						*School III*					
*Questions*		*III grade*		*V grade*		*p^a^-value*		*III grade*		*V grade*		*p^a^-value*		*III grade*		*V grade*		*p^a^-value*	
Mouth is an important part of the body																			
(a) True		76.1		87.0		0.331		83.5		89.5		0.279		89.8		83.1		0.404	
(b) False		14.7		6.5				9.8		4.2				4.2		6.2			
(c) Do not know		9.2		6.5				6.8		6.3				5.4		10.2			
Brushing your teeth twice daily can cause tooth decay																			
(a) True		11.9		8.1		0.649		2.3		3.2		0.716		12.7		3.1		0.09	
(b) False		84.4		90.3				94		94.7				85.6		92.3			
(c) Do not know		3.7		1.6				3.8		2.1				0.8		4.6			
Regular visit to the dentist helps to keep your teeth healthy																			
(a) True		91.7		95.2		0.661		89.5		91.6		0.866		86.4		80.0		0.244	
(b) False		6.4		3.2				6.8		5.3				7.6		9.2			
(c) Do not know		1.8		1.6				3.8		3.2				6.0		10.8			
Eating sweets, candies, and chocolates does not cause tooth decay																			
(a) True		8.3		3.2		0.321		3.8		1.10		0.265		10.2		4.6		0.263	
(b) False		90.8		96.8				94		98.9				87.3		93.8			
(c) Do not know		0.9		0				2.30		0				2.5		1.60			
Fizzy/carbonated drinks and frequent snacking are not good for teeth																			
(a) Yes		45.9		69.4		<0.001		44.4		56.8		0.173		40.7		60		0.014	
(b) No		35.8		8.1				24.8		14.7				35.6		27.7			
(c) Sometimes		13.8		11.3				17.3		13.7				5.1		7.7			
(d) I do not know		4.6		11.3				13.5		14.7				18.6		4.6			
Is it necessary to clean your tongue regularly?																			
(a) Yes		79.8		85.2		0.436		87.2		85.3		0.939		83.9		87.7		0.461	
(b) No		7.3		6.5				4.5		4.2				6.8		1.5			
(c) Sometimes		7.3		1.6				3.8		4.2				3.4		4.6			
(d) I do not know		5.5		6.5				4.5		6.3				5.9		6.2			
What does bleeding gums mean?																			
(a) Healthy gums		10.10		3.2		0.043		11.3		3.2		0.005		9.3		0		0.045	
(b) Clean gums		16.50		14.5				11.3		2.1				7.6		4.6			
(c) Gum disease		47.7		37.1				58.6		70.5				48.3		61.5			
(d) I do not know		25.7		45.2				18.8		24.2				34.7		33.8			
General health is related to oral health																			
(a)Yes		56		51.6		0.505		51.3		66.3		0.128		53.4		55.4		0.099	
(b) No		15.60		24.2				11.3		7.4				12.7		15.4			
(c) Sometimes		4.6		4.8				12.0		10.5				14.4		3.0			
(d) I do not know		23.9		19.4				25.6		15.8				19.5		26.2			
When should you visit your dentist?																			
(a) When in pain/discomfort only		52.3		43.5		0.318		68.4		52.6		0.029		62.7		61.5		0.210	
(b) Occasionally		5.50		12.9				5.3		2.1				3.4		7.7			
(c) Regularly every 6 months		35.8		38.7				23.3		42.1				16.1		23.1			
(d) I do not know		6.4		4.8				2.3		3.2				16.9		7.7			

^a^Chi-square test

In the Indian scenario, research on the association between age and dental caries has shown contradictory results. In the present study, there was no significant difference in dental caries status between the two age groups in all three schools. Rajesh and Venkatesh^10^ concluded that the highest prevalence of dental caries (49.7%) was seen in 6- to 10-year-old children followed by 11- to 15-year-olds (25.6%) in Tumkur district, Karnataka (South India). Kalaskar et al^11^ also showed an increase in the caries prevalence in the 6- to 10-year-olds when compared with 10- to 16-year-old Vidharba children of Central India. But, Kalita et al^12^ reported the prevalence of dental caries in 6- to 8-year-old Guwahati children (East India) to be 18.62% compared with 20.28% in 9- to 11-year-old children. The difference in the caries status of children across the country could be attributed to its vast and varied differences in cultural, ethnic and socioeconomic background.

There was a significant increase in dental caries treatment needs, as the caries status increased, in both third-grade and fifth-grade children. It is an apparent result that as dental caries status increased, their treatment needs also increased. Dhar and Bhatnagar^13^ had reported that 8- to 10-year-old children needed more treatment than the 6- to 7-year-olds. The older children were more in need of one surface filling, especially in first permanent molars and the younger children were in need of more sealants. In this study, caries treatment needs showed contradictory results in the three schools. Irrespective of the caries status, older children had a significant increase in dental caries treatment needs than the younger children in one school. When the caries subgroups were considered, in the no caries subgroup, older children needed more treatment. Since preventive restorations and fissure sealants were included as a part of the caries treatment, they accounted for the increase in treatment needs. But, as caries status increased, younger grade children needed more treatment than older grade children in another school. The younger children were in need of both preventive caries arresting care and conventional restorations.

Nur-E-Saud et al^14^ showed that lower grade children (6-8 years) had higher scores of debris index, calculus index, and OHI-S scores compared with higher grade children (11-14 years). In this study, significant difference in the OHI-S scores between third- and fifth-grade children was seen in two schools. In one school, third-grade children had relatively poor oral hygiene compared with the fifth-grade children. The operational abilities develop in a gradual and sequencing manner. As children grow, the simple skills are consolidated, combined, and reorganized into complex mental structures.^15^ Hence, older children are expected to perform oral hygiene measures effectively and regularly. In contrast, third-grade children of another school had better oral hygiene than the fifth-grade children. When the OHI-S scores were compared within the caries subgroups of the third- and fifth-grade children, similar contradictory results were obtained. Shift from parental supervision to personal care in carrying out oral hygiene measures could have also contributed for the same. The differences could be attributed to children’s attitude toward their oral health and their practice behavior in maintaining good oral hygiene. As dental caries status increased, the oral hygiene became significantly poor in third-grade children of one school. Chopra et al^16^ showed that children with high OHI-S scores had four times more risk of developing caries than those with good oral hygiene.

Considering overall knowledge scores, the older children had scored little better than the younger children in all the three schools. Acquisition of knowledge depends on child’s social interactions and experiences.^15^ Older children would have had more exposures, experiences, and interactions, which gave them more information on oral health than younger children. The dental caries status did not have any influence on the overall knowledge score, in both third- and fifth-grade children. School textbooks could have also played a major role in improving their oral health-related knowledge.^17^ Since information on general and oral health has become an integral part of the school syllabus, all children might have had the basic knowledge on oral health and its maintenance. Most of the third- and fifth-grade children were aware that brushing twice daily prevents dental caries; consuming sugared snacks, fizzy carbonated drinks, and frequent snacking causes tooth decay; regular tongue cleaning is necessary; mouth is an important part of the body; and that general health is related to oral health. Varenne et al^18^ reported that the majority of children knew that tooth cleaning and regular dental visits prevented oral disease. Piperakis et al^19^ reported that children were aware that regular dental visit was important, but only few of them had regular dental visits. Majority of the children in this study felt that the dentist should be visited only when there was pain or discomfort. Compared with the third-grade children, a higher proportion of fifth-grade children replied that dentist should be visited periodically every 6 months.

Piaget believed that all children go through a step-wise, consistent expansion of knowledge with age and he called it as an invariant developmental sequence.^8^ All children, irrespective of their culture, progressed through the same biologically programmed stages of development in a particular order. Children in this study belonged to concrete operational period with similar SES and cultural background. But, there was an inconsistency in results of the parameters assessed. This could have been due to the individual variations, their attitudes, and experiences. Their school syllabus provided them with knowledge on oral health, but did not have an effect on their attitude toward oral health or on their caries status. Children learn better through guided participation and interactive activity sessions rather than monologue lectures.^8^ This warrants the need for interactive school dental health education interventions with periodic reinforcements to enhance the oral health status of children in a holistic way. As this study was cross-sectional in nature, a causal relationship could not be established and only the associations between the parameters could be studied.

## CONCLUSION

In this study, age did not seem to influence the dental caries status, oral hygiene status, and knowledge related to oral health. Both the younger and older children had similar caries status. Contradictory results were obtained in respect to caries treatment needs and oral hygiene status. Third-grade children of one school and the fifth-grade children of another school had poor oral hygiene and higher treatment needs. Though older children are more mature cognitively, the attitude to put knowledge into practice was lacking.
